# A Cross-Sectional Evaluation of Disability in Inflammatory Bowel Disease Using IBD Disk in a Tertiary Center from Romania

**DOI:** 10.3390/jcm13237168

**Published:** 2024-11-26

**Authors:** Oana-Maria Muru, Corina Silvia Pop, Petruța Violeta Filip, Nicoleta Tiucă, Laura Sorina Diaconu

**Affiliations:** 1 Department of Internal Medicine 2 and Gastroenterology, Bucharest University Emergency Hospital, 050098 Bucharest, Romania; oana-maria.neagu@drd.umfcd.ro (O.-M.M.); corina-pop@umfcd.ro (C.S.P.); nicoltiuca@gmail.com (N.T.); sorina.diaconu@umfcd.ro (L.S.D.); 2 Carol Davila University of Medicine, 050474 Bucharest, Romania

**Keywords:** Crohn’s disease, ulcerative colitis, inflammatory bowel diseases, disability

## Abstract

**Background/Objectives**: The management of inflammatory bowel disease (IBD) includes, besides the control of symptoms, the prevention of organ damage and the improvement of the overall disability. **Methods**: A single-centered, cross-sectional, non-interventional and population-based study was conducted between October 2023 and August 2024 in the Department of Internal Medicine 2 and Gastroenterology of Bucharest Emergency University Hospital to assess the disease disability and quality of life impact using IBD-disk and correlation with different parameters. **Results**: We included 112 patients; their mean age was 52.35 ± 16.67 years, with a disease duration of 114.9 ± 97.93 months. The majority of patients were represented by men (51.79%). We observed a strong correlation between the CDAI score and overall disability compared to the Mayo score for UC (*p* = 0.0068). Also, patients with CD and stenotic patterns, as well as the presence of extraintestinal complications, have associated high disability scores. Low hemoglobin levels are associated with high disability (*p* = 0.0164), while biological treatment is associated with low disability (*p* = 0.0481). **Conclusions**: IBD-disk can be used as a valuable tool to assess disability in patients with IBD, also in terms of the activity of the disease, but mostly in terms of the psychological burden of the disease.

## 1. Introduction

Inflammatory bowel diseases (IBDs), represented by Crohn’s disease (CD) and ulcerative colitis (UC), are characterized by an immune-mediated chronic inflammation of the gastrointestinal tract [1,2]. The general prevalence of IBD is increasing, with North America and Europe having the highest reported IBD rates [1,2,3]. Since most of the patients are young and active in the work-field, this makes the work disability a major impairment and also adds a significant burden on the healthcare system [4,5]. The European Crohn’s and Ulcerative Federation conducted a survey on 4670 patients with IBD, showing that more than 50% are affected by the lack of available toilets, which leads to absenteeism at work and career harm, and 35% of them cannot have intimate relationships [6]. “Normal life” is a dream for patients with IBD, and the prevention of disability is now our major therapeutic goal [7,8].

Over the past three decades, the public health issue of disability has been increasingly addressed so that the management of IBD has shifted from solely controlling symptoms to achieving the prevention of organ damage and disability [3,5]. The clinical picture includes remission alternating with flares or adverse reactions to medical treatment or surgical interventions, all of which lead to diminished physical and psychological well-being, productivity, and even family and social isolation [4].

Since 2007, the IBD Disability Index (IBD-DI) has been used by physicians to assess the disability of patients with IBD [3,5,9,10,11]. However, the complexity of this questionnaire makes its use deficient if clinicians lack the time to administer it at every visit [12].

In 2020, the VALIDATE study showed that the IBD Disk is an easy tool that can be used in daily practice to assess disability in patients with IBD [12]. Sometimes, the lack of medical education and local socio-cultural influences can make IBD disk application difficult, as noted by Singh et al. in their article [13].

In general, IBD affects young people, and often, the impact of the disease is also reflected in their sex life, leading not only to stigma but also to increased levels of anxiety and depression, which often leads to poor control of the disease [2,14,15,16]. The identification of anxiety and depression is still the responsibility of the gastroenterologist, who must identify these disorders and, together with the psychologist/psychiatrist, intervene and improve the patient’s well-being [14,15,16]. However, Romanian patients are reticent about psychological/psychiatric counselling and accept their intervention with difficulty.

In our clinic, the clinician’s time often does not allow for proper assessment of IBD-related disability. We aimed to apply the IBD disk among our patients to correlate disease activity and associated disability and to analyze correlations between clinical and paraclinical parameters, associated comorbidities and disability scores in patients with different socio-cultural principles from Western Europe.

## 2. Materials and Methods

### 2.1. Study Design, Protocol and Patients

This single-center, cross-sectional, non-interventional, and population-based study was conducted at the Department of Internal Medicine 2 and Gastroenterology of the Bucharest Emergency University Hospital, Romania.

Patients with IBD were recruited from November 2023 to August 2024. Both inpatients and outpatients completed the questionnaire, which was translated into their native language. The study included participants who achieved clinical remission and those who had symptoms of active disease.

The study was conducted following the principles of the Declaration of Helsinki. The protocol was approved by the Local Ethics Committee of the Bucharest Emergency University Hospital (protocol No. 55965 of 1 November 2023).

All patients signed an informed consent confirming their willingness to participate in the study. The inclusion criteria were (1) the diagnosis of IBD according to European Crohn’s and Colitis Organization (ECCO) guidelines; (2) adult population >18 years; (3) complete biological panel; and (4) signed informed consent. The exclusion criteria were (1) patients with nonspecific colitis; (2) acute severe infections (e.g., septic shock); (3) end-stage chronic diseases (e.g., stage IV heart failure, stage IV chronic obstructive pulmonary disease (COPD)); (4) metastatic cancer; (5) admission to the intensive care unit.

The data were collected from the hospital’s electronic records. The following variables were included in the study: demographics (age, sex), anthropometry (height, weight, body mass index—BMI), risk factors (smoking history), comorbidities, history of infections (tuberculosis, Clostridium difficile, cytomegalovirus), clinical features (duration of IBD, extension for ulcerative colitis: E1-proctitis, E2-left colitis, E3-pancolitis and for Crohn’s disease: ileal disease—L1, colonic disease—L2, ileo-colonic disease—L3, upper gastrointestinal tract—L4), activity scores: Mayo for ulcerative colitis and CDAI for CD, disease behavior, history of surgery, presence of extraintestinal manifestations and intestinal complications, as well as current treatment (5-ASA, steroids, azathioprine, biological therapy). Biochemical parameters, including serum C-reactive protein (CRP), hemoglobin (Hb), fecal calprotectin (FC) and albumin, were also included. The baseline was considered to be at the initial disability assessment using the IBD disk.

To assess disability in IBD, we used the IBD Disability Disk (with the approval and courtesy of Professor Subrata Ghosh). The questionnaire was applied at the hospital, and when it was impossible, an online form was used. It included the 10 items selected from the IBD Disability Index—abdominal pain, body image, education and work, emotions, energy, interpersonal interactions, joint pain, defecation regulation, sleep and sexual function. The IBD-Disk is particularly useful, easy to administer on an outpatient basis, and provides a quick representation of the level of disability. Each component is scored from 0 to 10, where 0 means no disability and 10 means maximum disability. The total IBD disk score was calculated as the sum of the 10 individual component subscores (minimum score 0 and maximum score 100). A score above 5 is considered a high burden.

### 2.2. Statistical Analysis

Statistical analysis included descriptive statistics (frequency, percentage, mean, median, standard deviation) and inferential statistics. The Shapiro–Wilk test was applied to determine the distribution of the analyzed data series. The Mann–Whitney non-parametric test for comparing medians in non-Gaussian distributions was applied. The Spearman non-parametric test was applied to non-Gaussian distributed data series to assess the correlation (to measure the strength of association) between quantitative variables. The significance threshold chosen for the *p*-value was 0.05. The internal consistency of the IBD-disk questionnaire was assessed using the Cronbach coefficient alpha (α). Cronbach’s alpha > 0.7 and corrected item-total correlation > 0.4 were considered significant. Statistical analysis was performed using the SPSS Statistics utility, trial version 29.0.0.

## 3. Results

### 3.1. Group Characteristics

**Figure 1:**
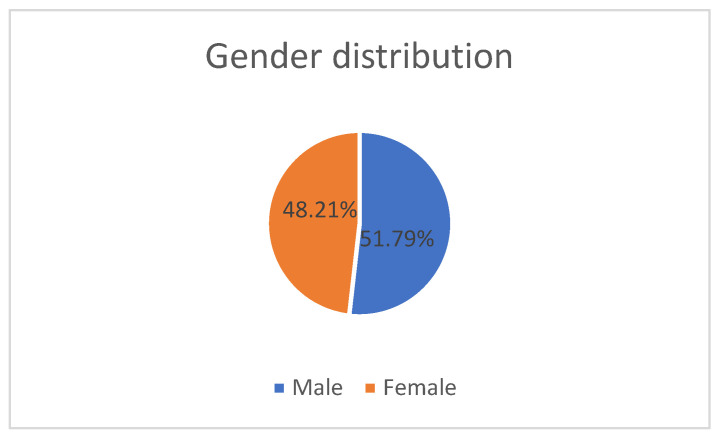
Gender distribution of the patients with IBD.

A total of 112 patients with IBD with a mean age of 52.35 ± 16.67 (51.00) were enrolled. The male gender was the majority (51.79%) in our study group, with a disease distribution of 62.50% UC and 37.50% CD and a mean disease duration of 114.9 ± 97.93 months (Table 1, Figure 1).

Assessing the impact of risk factors in chronic disease is extremely important in IBD, and especially in CD, as smoking is associated with a higher risk of flares and surgical complications, contributing to overall disability. In our study, most were non-smokers (61.61%), and only 25% were active smokers (Table 1). Often, associated infections can influence the course and severity of the disease, causing disability. Patients included in the study had tuberculosis (15.18%) and clostridium infections (20.54%) (Table 1).

In terms of disease extent, among the 70 patients with UC, most had left-sided colitis (41.43%), and 38.57% had pancolitis-E3 (Table 1). Regarding patients with CD, in our study, half had ileo-colonic disease, 26.19% colonic disease, and 26.19% ileal disease (Table 1). Regarding Crohn’s disease behavior, 54.76% had inflammatory disease (nonpenetrating nonstrictive), 35.71% stenotic disease (stenotic), and 9.52% fistulous disease (penetrating) (Table 1). A small proportion of patients (9.82%) had intestinal complications, such as intestinal fistulas (four patients), intestinal obstruction (three patients), or abscesses (three patients) (Table 1). Nineteen patients required surgery according to their medical history (Table 1).

The CDAI and Mayo scores assessed disease severity inpatients with Crohn’s and UC. The mean CDAI score was 249.5 ± 132.2, meaning that most patients with Crohn’s disease had active disease (Table 1).

The treatment options varied. Only 13.64% (15 patients) achieved disease control with 5-ASA, and 19.64% (22 patients) received immunosuppressive treatment (corticosteroids, methotrexate, or azathioprine, ± 5-ASA) (Table 1). Most of the patients received biologic therapy (48.21%), and 16.96% (19 patients) received combinations of biologic and immunosuppressive therapy (Table 1).

**Figure 2:**
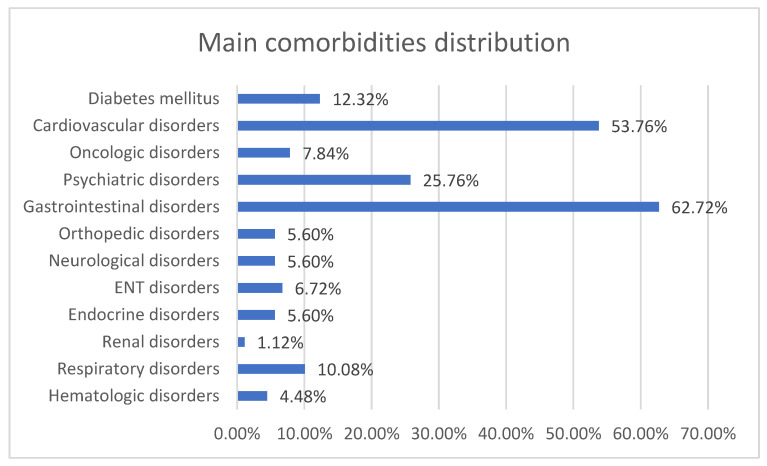
Main comorbidity distribution of patients with IBD.

The patients included in the study also presented comorbidities such as other gastrointestinal disorders (67.72%), cardiovascular disorders (53.76%), and psychiatric disorders (25.76%) (Figure 2).

### 3.2. Influence of Different Parameters in IBD Disk Scores

We compared different parameters with the scores obtained in the IBD disk using a series of statistical tests, and we followed their impact on the quality of life of the patient with IBD.

After statistical analysis of age, gender, type of inflammatory bowel disease, disease extension and total IBD-disk score, we did not obtain statistically significant data. However, younger, active patients presented higher disability scores regarding interpersonal interactions, energy and body image despite being considered in remission. In Crohn’s disease, we observed a strong correlation between a high disease activity score (CDAI) and IBD disk (*p* = 0.006), which was not observed in patients with ulcerative colitis (Table 2). CD phenotype was associated with high disability scores in patients who presented with stenotic forms of disease versus those with an inflammatory pattern (*p* = 0.0197) (Table 3). In both diseases, an intestinal complication is associated with high disability scores (*p* = 0.0116) (Table 4). Cronbach alpha applied in our study for the entire IBD questionnaire was 0.947, with excellent reliability (internal consistency).

Over the years, we have noticed that patients with IBD presented many comorbidities that also influenced their quality of life. Using anthropometric measures, we observed that our patients with IBD suffer from obesity. And most of them were also diagnosed with metabolic syndrome. These pathologies were associated with significant statistical value with high disability scores (Table 5 and Table 6).

We wanted to see any correlation between inflammation biomarkers such as CRP, ESR, FC, low albumin value, and IBD disk values. Still, we did not obtain statistically significant results. We obtained a significant correlation between hemoglobin values and total IBD disk score, which means low hemoglobin values are associated with high IBD disk score values (*p* = 0.0164) (Table 7). Patients with advanced therapies, such as biological treatment, present low values for disability scores (*p* = 0.0481) (Table 8).

At univariable analysis for IBD disk total scores, we found the following variables as significant statistical predictor factors: BMI (*p* = 0.0020), obesity (*p* = 0.0104), metabolic syndrome (*p* = 0.0124.) and biological parameters like hemoglobin (*p* = 0.0032), ESR (*p* = 0.0095) and fecal calprotectin (*p* = 0.0124) (Table 9). At multivariable analysis, we found that 12.4% of the variation in IBD disk total score values was due to the predictor variables: BMI, Type of Disease, Disease duration (months), Gender, and Age, and the regression model was statistically significant (*p* = 0.0049). In addition, 11.5% were due to the predictor variables, biological parameters and treatment, and the regression model was statistically significant (*p* = 0.0055). As for the biological parameters, in the multivariable regression model, for every 1-unit increase in hemoglobin values, the IBD disk total score value will decrease by 2.510, which is statistically significant. Regarding the treatment, for every 1-unit increase in treatment (from No treatment to 5-ASA to Immunosuppressive treatment to biological treatment to Biological and immunosuppressive treatment), the IBD disk total score value will decrease by 4.794, which is statistically significant.

## 4. Discussion

The average age of our patients was 52 years, which means they are an essential part of active society. However, in our study, age was correlated with a higher burden for work and education, as well as for sexual function, which means that the disease (both in remission and during flares) brings an additional burden on these patients, which affects their life at work, school or home. In the literature, most of the time, patients with IBD are young, with an active social and professional life [2,3].

This short form of the IBD disability questionnaire successfully brought new information to our study—high scores for interpersonal interactions, energy, and body image were recorded in young, active patients who were in remission. Due to these values, global disability was high despite low disease activity, which means that the IBD disk may be a valuable tool for assessing disability beyond well-known clinical and paraclinical parameters.

Studies in the literature have shown that higher total IBD disk scores were observed in patients with higher disease activity scores [2,12,17]. Age, duration of the disease, phenotype and extension of the disease did not affect the disability scores [2,13,17]. Our study obtained strong correlations between CDAI score and overall disability, more substantial than the Mayo score for patients with UC (*p* = 0.0068), similar to those in the literature. Moreover, the CD phenotype associates high disability scores in patients with stenotic forms of the disease compared to those with an inflammatory pattern (*p* = 0.0197). High disability scores were also correlated with the presence of intestinal complications in both types of disease (*p* = 0.0116). Both diseases are associated with a lack of energy following IBD disk application, but in our study, patients with Crohn’s disease have a more significant energy burden compared to patients with UC, perhaps due to the greater need for surgery, but also to the greater frequency of complications in CD [2,13,17]. Furthermore, these patients were also associated with significant body image anxiety, especially in those patients with strictures and penetrating disease. This phenotype of the disease is frequently associated with an increased need for extensive, mutilating surgical interventions [18,19].

What our study adds is that obesity and metabolic syndrome, two comorbidities associated with IBD, negatively influence IBD disk scores. Obesity and metabolic syndrome influenced patients’ emotions, social interactions, access to education/work, and sleep. In both univariate and multivariate analysis, these two comorbidities statistically significantly influence total IBD disk scores from the statistical point of view. Weight loss should be a primary goal, with a role in reducing systemic inflammation, joint pain and cardiovascular comorbidities (which have a high prevalence in IBD patients) [20]. Obese patients with IBD are usually associated with sarcopenia, which is secondary to inflammation, malnutrition, and intestinal dysbiosis, thus requiring additional attention regarding dietary and physical interventions to increase muscle mass and decrease adiposity [6,10,21,22].

In our patients with IBD, low hemoglobin places a high burden on interpersonal interactions, energy, and joint pain, which means that treating anemia may improve overall disability and should be another important target for managing patients with IBD. It is well-known that anemia is associated with fatigue, low energy level and overall disability [23,24]. Anemia is “an often-overlooked extraintestinal manifestation in IBD” that adversely affects the patient’s quality of life, physical performance levels, concentration levels, and cognitive functioning [25]. Untreated anemia can lead to extreme fatigue, heart failure, and depression [23,26]. Until now, no study in this field has considered the impact of anemia on IBD-disk scores.

A better control of inflammation using newer, advanced therapies leads to clinical, endoscopic, and mucosal remission, a reduced number of intra- and extra-intestinal complications, and a reduced risk of developing new comorbidities [1,11,18,27,28,29]. Our study notes that biological treatment was associated with a reduced risk of disability (*p* = 0.0481). In addition, correcting the hemoglobin level, increasing muscle mass, and reducing obesity improve patients’ well-being. The results of our study show that even if we obtain remission of the disease using new therapies, we often neglect the early treatment of complications/comorbidities like anemia, obesity, or metabolic syndrome.

Two of the most critical targets in the management of the disease, according to the STRIDE-II guidelines, are to improve the quality of life and the absence of disability in IBD [1]. Usually, the assessment of disease activity takes into account several parameters (clinical, biological, endoscopic, histological) without including the assessment of disability, such as body image, energy level, social interactions or sexual functions [25,30].

The IBD disk has demonstrated its value and utility in large patient cohorts, but the profile of patients is different depending on the socio-economic culture of each country [13,17]. Another problem is that our patients lack medical education and sometimes have limited access to medical services due to a lack of medical insurance. All these problems cause a delay in diagnosis and the association of intestinal and extraintestinal complications that lead to difficult disease management and, implicitly, to disability.

One of the limitations of our study was that it took place in a single center and included a small number of patients with IBD. Another limitation was represented by the more significant number of patients with ulcerative colitis, who were mainly in remission compared to those with CD, who had mild to moderate disease activity. Moreover, we were faced with the reluctance of these patients when completing the questionnaire due to their low levels of education and high levels of stigma. This was conducted to achieve our secondary objective: to collect the IBD disk scores at six months from baseline.

## 5. Conclusions

In conclusion, the IBD disk is a valuable tool for assessing IBD-related disability and promoting discussion of the patient’s daily burden. It also challenges the gastroenterologist to find an appropriate solution that includes physical, dietary, and psychological/psychiatric interventions to improve the quality of life. Moreover, periodic disability assessment can lead to early identification of a possible disease relapse.

## Figures and Tables

**Table 1:** The general characteristics of the patients included in the study—clinical and biological features, phenotype, extension and complications of the disease and treatment.

Characteristics	Number of Patients = 112 (Value)	
Age (years)/Mean ± SD (Median)		52.35 ± 16.67 (51.00)
Gender/Frequency (%)	Males/Females	58 (51.79%)/54 (48.21%)
Disease duration (months)/Mean ± SD (Median)		114.9 ± 97.93 (96.00)
Type of inflammatory bowel disease/(%)	UC/CD	70 (62.50%)/42 (37.50%)
Anthropometric	BMI	24.02 ± 4.571 (23.38)
Smoking	Non-smoker/Smoker/Former smoker	69 (61.61%)/28 (25.00%)/15 (13.39%)
Infections	Tuberculosis	17 (15.18%)
	Clostridium difficile	23 (20.54%)
Biologic parameters	Hb (g/dL)	12.48 ± 1.967 (12.80)
	CRP (mg/L)	6.398 ± 17.59 (1.750)
	ESR (mm/h)	27.23 ± 17.87 (23.00)
	Albumin (g/dL)	3.739 ± 0.7414 (3.65)
	Ferritin (ug/L)	107.2 ± 92.37 (71.00)
	FC (ug/g)	470.6 ± 1105 (98.00)
History of surgery		19 (16.96%)
Treatment/Frequency (%)		110 (98.21%)
5-ASA		15 (13.64%)
Immunosuppressive treatment		22 (19.64%)
Biologic treatment ± 5-ASA		54 (48.21%)
Biologics + Immunosuppressants ± 5-ASA		19 (16.96%)
UC Disease extent/Frequency (%)		
E1		14 (20.00%)
E2		29 (41.43%)
E3		27 (38.57%)
CD Disease extent/Frequency (%)		
Ileal disease		11 (26.19%)
Colonic disease		8 (19.05%)
Ileocolonic disease		21 (50.00%)
Other		2 (4.76%)
Disease Phenotype		
Non-penetrating, non-structuring disease		23 (54.76%)
Structuring disease		15 (35.71%)
Penetrating disease		4 (9.52%)
Intestinal complications		
Intestinal fistulas		4(36.36%)
Bowel obstruction		3(27.27%)
Abscesses		3 (27.27%)
Lower intestinal bleeding		1(9.10%)
Activity disease		
CDAI		249.5 ± 132.2 (237.0)
Mayo score		2.786 ± 1.676 (3.00)

CD—Crohn’s disease, UC—ulcerative colitis, BMI—body mass index, Hb—hemoglobin, CRP—C-reactive protein, FC—fecal calprotectin, 5-ASA—5 aminosalicylates, E1—rectitis, E2—left-sided colitis, E3—Extensive UC (pancolitis), CDAI—Crohn’s Disease Activity Index.

**Table 2:** Spearman correlation between activity disease score—CDAI and IBD disk scores.

	CDAI score (n = 42)		
	r Coefficient	95% Confidence Interval	*p*-Value
Abdominal pain	0.3642	0.05849 to 0.6075	0.0177
Regulating defecation	0.2766	−0.03922 to 0.5421	0.0762
Interpersonal interactions	0.4398	0.1477 to 0.6613	0.0036
Education and work	0.4339	0.1405 to 0.6572	0.0041
Sleep	0.2533	−0.06418 to 0.5242	0.1055
Energy	0.3596	0.05321 to 0.6042	0.0193
Emotions	0.3627	0.05671 to 0.6064	0.0182
Body image	0.4373	0.1447 to 0.6596	0.0038
Sexual functions	0.3330	0.02299 to 0.5846	0.0312
Joint pain	0.1850	−0.1352 to 0.4702	0.2408
IBD-disk total score	0.4114	0.1136 to 0.6414	0.0068

**Table 3:** Spearman correlation Crohn’s disease phenotype and IBD disk scores.

Dunn’s Multiple Comparison Test	*p*-Value
N-penetrating N-structuring vs. Structuring disease	0.0197
N-penetrating N-structuring vs. Penetrating disease	0.0870
Structuring disease vs. Penetrating disease	0.7606

**Table 4:** Spearman correlation between the presence of intestinal complications and IBD disk scores.

	With Intestinal Complications (n = 74) Mean ± SD (Median)	Without Intestinal Complications (n = 38) Mean ± SD (Median)	*p*-Value
Abdominal pain	2.905 ± 2.685 (2.000)	2.816 ± 3.384 (1.000)	0.2087
Regulating defecation	2.432 ± 2.532 (1.500)	2.763 ± 3.420 (0.500)	0.6308
Interpersonal interactions	3.932 ± 3.159 (3.500)	2.263 ± 2.777 (1.000)	0.0049
Education and work	3.946 ± 3.043 (4.000)	2.132 ± 2.859 (1.000)	0.0031
Sleep	4.365 ± 2.870 (4.500)	3.158 ± 3.460 (1.500)	0.0270
Energy	4.959 ± 3.027 (5.000)	3.579 ± 3.382 (2.000)	0.0221
Emotions	4.703 ± 3.170 (6.000)	3.421 ± 3.607 (1.500)	0.0427
Body image	4.473 ± 3.211 (5.500)	2.237 ± 2.716 (1.000)	0.0003
Sexual functions	2.622 ± 2.642 (2.000)	1.211 ± 2.473 (0.000)	0.0004
Joint pain	4.014 ± 2.620 (4.000)	3.632 ± 3.664 (2.000)	0.2717
IBD-disk total score	38.84 ± 24.42 (40.00)	27.03 ± 25.24 (17.00)	0.0116

**Table 5:** Spearman correlations between obesity and IBD disk scores.

Comorbidities	With Obesity (n = 34) Mean ± SD (Median)	Without Obesity (n = 78) Mean ± SD (Median)	*p*-Value
Abdominal pain	1.882 ± 2.319 (1.000)	3.308 ± 3.068 (2.000)	0.0200
Regulating defecation	2.294 ± 2.887 (1.000)	2.654 ± 2.851 (2.000)	0.4801
Interpersonal interactions	2.353 ± 2.891 (1.500)	3.808 ± 3.138 (4.000)	0.0218
Education and work	2.353 ± 2.740 (2.000)	3.756 ± 3.155 (3.500)	0.0386
Sleep	2.971 ± 3.060 (2.000)	4.385 ± 3.067 (5.000)	0.0258
Energy	3.706 ± 3.205 (2.000)	4.833 ± 3.164 (5.000)	0.0775
Emotions	3.206 ± 3.365 (1.500)	4.731 ± 3.278 (6.000)	0.0291
Body image	2.912 ± 3.397 (2.000)	4.064 ± 3.097 (4.000)	0.0580
Sexual functions	1.294 ± 2.140 (0.000)	2.513 ± 2.790 (2.000)	0.0215
Joint pain	3.118 ± 3.073 (2.000)	4.218 ± 2.931 (4.000)	0.0626
IBD-disk total score	25.68 ± 21.71 (18.50)	38.82 ± 25.73 (43.00)	0.0209

**Table 6:** Spearman correlation between metabolic syndrome and IBD disk scores.

	With Metabolic Syndrome (n = 32) Mean ± SD (Median)	Without Metabolic Syndrome (n = 80) Mean ± SD (Median)	*p*-Value
Abdominal pain	1.813 ± 2.250 (1.000)	3.300 ± 3.066 (2.000)	0.0189
Regulating defecation	1.594 ± 2.421 (0.000)	2.925 ± 2.937 (2.000)	0.0159
Interpersonal interactions	2.281 ± 2.738 (2.000)	3.800 ± 3.180 (4.000)	0.0249
Education and work	2.281 ± 2.774 (1.500)	3.750 ± 3.128 (3.000)	0.0215
Sleep	2.938 ± 2.711 (2.000)	4.363 ± 3.195 (5.000)	0.0358
Energy	3.688 ± 2.741 (3.000)	4.813 ± 3.334 (5.000)	0.1091
Emotions	2.906 ± 3.020 (2.000)	4.813 ± 3.357 (6.000)	0.0064
Body image	3.125 ± 3.139 (2.000)	3.950 ± 3.241 (4.000)	0.2136
Sexual functions	1.344 ± 1.860 (0.000)	2.463 ± 2.868 (1.500)	0.1009
Joint pain	3.281 ± 2.785 (3.000)	4.125 ± 3.070 (4.000)	0.2011
IBD-disk total score	25.66 ± 20.34 (17.00)	38.50 ± 26.15 (40.00)	0.0202

**Table 7:** Spearman correlation between hemoglobin level and IBD disk scores.

	Hemoglobin (n = 112)		
	r Coefficient	95% Confidence Interval	*p*-Value
Abdominal pain	−0.1603	−0.3408 to 0.03161	0.0913
Regulating defecation	−0.1766	−0.3556 to 0.01484	0.0625
Interpersonal interactions	−0.1920	−0.3694 to −0.001086	0.0426
Education and work	−0.2577	−0.4276 to −0.07021	0.0061
Sleep	−0.1651	−0.3452 to 0.02666	0.0819
Energy	−0.2317	−0.4047 to −0.04263	0.0140
Emotions	−0.1342	−0.3170 to 0.05827	0.1584
Body image	−0.1618	−0.3421 to 0.03013	0.0884
Sexual functions	−0.1770	−0.3559 to 0.01445	0.0619
Joint pain	−0.2320	−0.4051 to −0.04300	0.0138
IBD-disk total score	−0.2263	−0.4000 to −0.03699	0.0164

**Table 8:** Spearman correlations between biological treatment and IBD disk scores.

	With Biologic Treatment (n = 73) Mean ± SD (Median)	Without Biologic Treatment (n = 39) Mean ± SD (Median)	*p*-Value
Abdominal pain	2.685 ± 2.999 (2.000)	3.231 ± 2.786 (2.000)	0.1211
Regulating defecation	2.438 ± 3.005 (1.000)	2.744 ± 2.572 (2.000)	0.1944
Interpersonal interactions	2.890 ± 3.085 (2.000)	4.256 ± 3.041 (4.000)	0.0145
Education and work	2.932 ± 3.106 (2.000)	4.077 ± 2.959 (4.000)	0.0445
Sleep	3.575 ± 3.261 (3.000)	4.667 ± 2.737 (6.000)	0.0455
Energy	4.260 ± 3.342 (4.000)	4.923 ± 2.923 (5.000)	0.2572
Emotions	3.959 ± 3.529 (3.000)	4.846 ± 2.987 (6.000)	0.1369
Body image	3.233 ± 3.243 (2.000)	4.615 ± 3.014 (5.000)	0.0209
Sexual functions	1.767 ± 2.617 (0.000)	2.846 ± 2.631 (2.000)	0.0034
Joint pain	3.863 ± 3.216 (4.000)	3.923 ± 2.599 (4.000)	0.6846
IBD-disk total score	31.60 ± 25.29 (21.00)	40.87 ± 24.28 (51.00)	0.0481

**Table 9:** Results from univariate analysis that uses univariable independent factors that could influence the IBD scores and multivariable regression that uses multiple factors that could influence the IBD scores.

Variable	Univariable			Multivariable		
	Unstandardized Coefficients B	95% CI	*p*-Value	Unstandardized Coefficients B	95% CI	*p*-Value
Characteristics						
Age	−0.300	−0.609 to 0.009	0.0568	−0.156	−0.466 to 0.154	0.3195
Gender	−9.936	−20.103 to 0.230	0.0553	−9.887	−19.895 to 0.121	0.0527
Disease duration (months)	0.025	−0.094 to 0.006	0.0858	−0.041	−0.091 to 0.010	0.1134
Type of Disease	5.725	−5.808 to 17.259	0.3267	2.312	−8.681 to 13.305	0.6770
BMI	−1.729	−2.812 to −0.646	0.0020	−1.368	−2.465 to −0.270	0.0151
Comorbidities						
Diabetes	−3.504	−18.345 to 11.338	0.6399	3.309	−12.541 to 19.159	0.6788
IC	−7.913	−21.484 to 5.659	0.2495	−1.729	−17.550 to 14.093	0.8284
High Blood Pressure	−7.917	−18.871 to 3.036	0.1543	−1.561	−15.371 to 12.249	0.8225
Obesity	−14.382	−25.305 to −3.458	0.0104	−9.250	−23.030 to 4.530	0.1853
Metabolic Syndrome	−14.504	−25.797 to −3.211	0.0124	−8.314	−23.922 to 7.294	0.2923
Biologic parameters						
Hemoglobin	−3.540	−5.869 to −1.212	0.0032	−2.510	−4.992 to −0.027	0.0475
C-reactive protein	0.173	−0.096 to 0.442	0.2049	0.003	−0.292 to 0.298	0.9833
ESR	0.344	0.086 to 0.603	0.0095	0.234	−0.064 to 0.532	0.1223
Albumin	−3.072	−9.473 to 3.330	0.3437	−0.705	−7.160 to 5.751	0.8290
Ferritin	−0.002	−0.053 to 0.050	0.9527	0.006	−0.046 to 0.057	0.8328
Fecal calprotectin	0.005	0.001 to 0.010	0.0124	0.003	−0.001 to 0.008	0.1748
Treatment	−3.930	−7.910 to 0.049	0.0528	−4.794	−8.678 to −0.910	0.0160

## Data Availability

The data that support the findings of this study are available from the corresponding author upon reasonable request.
